# 2-Azido-1-(4-methyl­phen­yl)ethanone

**DOI:** 10.1107/S1600536812018491

**Published:** 2012-05-05

**Authors:** Muhammad Arshad, Sammer Yousuf, Hafiza Madiha Butt, Sumayya Saeed, Fatima Z. Basha

**Affiliations:** a H.E.J. Research Institute of Chemistry, International Center for Chemical and Biological Sciences, University of Karachi 75270, Pakistan; b Department of Chemistry, University of Karachi 75270, Pakistan

## Abstract

In the mol­ecule of the title compound, C_9_H_9_N_3_O, the angle formed by the least-squares line through the azide group with the normal to the plane of the benzene plane ring is 46.62 (16)°. The crystal structure features C—H⋯O hydrogen bonds, which link the mol­ecules into zigzag chains running parallel to [010].

## Related literature

For a related structure, see: Yousuf *et al.* (2012 ▶). For the biological activity of triazoles, see: Genin *et al.* (2000 ▶); Parmee *et al.* (2000 ▶); Koble *et al.* (1995 ▶); Moltzen *et al.* (1994 ▶).
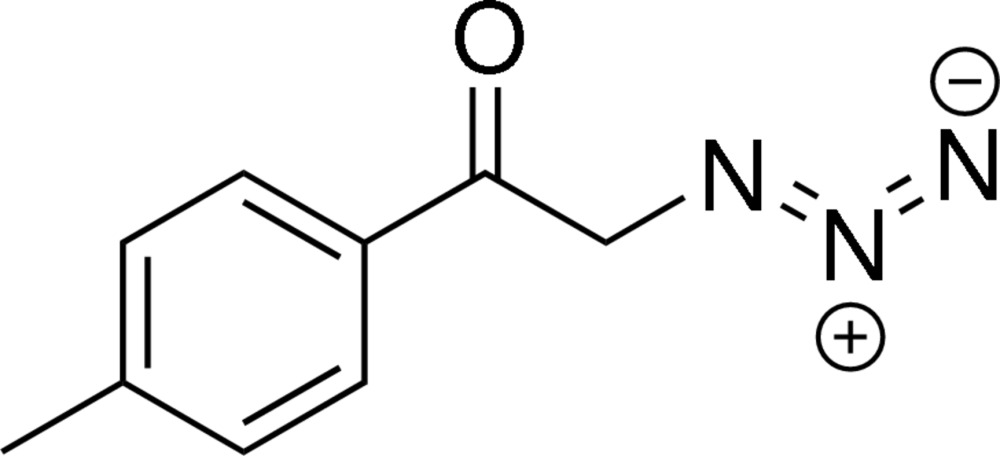


## Experimental

### 

#### Crystal data

C_9_H_9_N_3_O
*M* *_r_* = 175.19
Monoclinic, 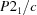

*a* = 7.696 (3) Å
*b* = 9.025 (3) Å
*c* = 14.248 (4) Å
β = 118.726 (15)°
*V* = 867.8 (5) Å^3^
*Z* = 4
Mo *K*α radiation
μ = 0.09 mm^−1^
*T* = 273 K
0.30 × 0.21 × 0.17 mm

#### Data collection

Bruker SMART APEX CCD area-detector diffractometer
Absorption correction: multi-scan (*SADABS*; Bruker, 2000 ▶) *T*_min_ = 0.973, *T*_max_ = 0.985
4915 measured reflections
1595 independent reflections
1464 reflections with *I* > 2σ(*I*)
*R*_int_ = 0.019

#### Refinement

*R*[*F* ^2^ > 2σ(*F* ^2^)] = 0.034
*wR*(*F* ^2^) = 0.090
*S* = 1.07
1595 reflections
120 parameters
H-atom parameters constrained
Δρ_max_ = 0.24 e Å^−3^
Δρ_min_ = −0.28 e Å^−3^

### 

Data collection: *SMART* (Bruker, 2000 ▶); cell refinement: *SAINT* (Bruker, 2000 ▶); data reduction: *SAINT*; program(s) used to solve structure: *SHELXS97* (Sheldrick, 2008 ▶); program(s) used to refine structure: *SHELXL97* (Sheldrick, 2008 ▶); molecular graphics: *SHELXTL* (Sheldrick, 2008 ▶); software used to prepare material for publication: *SHELXTL*, *PARST* (Nardelli, 1995 ▶) and *PLATON* (Spek, 2009 ▶).

## Supplementary Material

Crystal structure: contains datablock(s) global, I. DOI: 10.1107/S1600536812018491/rz2744sup1.cif

Structure factors: contains datablock(s) I. DOI: 10.1107/S1600536812018491/rz2744Isup2.hkl

Supplementary material file. DOI: 10.1107/S1600536812018491/rz2744Isup3.cml

Additional supplementary materials:  crystallographic information; 3D view; checkCIF report

## Figures and Tables

**Table 1:** Hydrogen-bond geometry (Å, °)

*D*—H⋯*A*	*D*—H	H⋯*A*	*D*⋯*A*	*D*—H⋯*A*
C8—H8*A*⋯O1^i^	0.97	2.40	3.2404 (19)	145

Symmetry code: (i) 
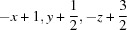
.

**Fig. 1.**
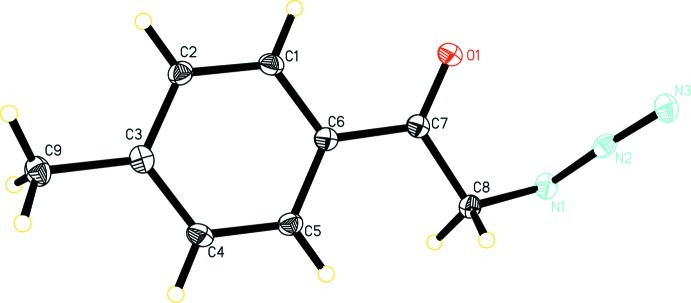
The molecular structure of the title cmpound with displacement ellipsoids drawn at the 30% probability level.

**Fig. 2.**
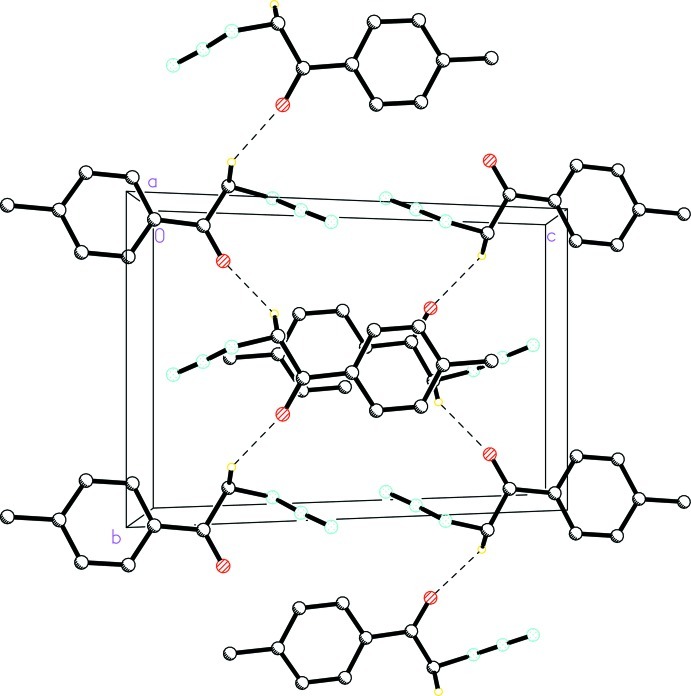
Crystal packing of the title compound viewed along the a axis. Hydrogen atoms not involved in hydrogen bonds (dashed lines) are omitted for clearity.

## supplementary crystallographic information

### Comment

Triazoles are considered an important class of compounds due to their therapeutic potential (Genin *et al.*, 2000; Parmee *et al.*, 2000; Koble *et al.*, 1995; Moltzen *et al.*, 1994). The title compound was obtained as an intermediate during our attempt to synthesize biologically active triazoles.

The structure of the title compound (Fig. 1) is similar to that of our recently published compound 2-azido-1-(4-fluorophenyl)ethanone (Yousuf *et al.*, 2012) with the difference that the fluorophenyl ring is replaced by a toluene ring. The bond lengths and angles are similar to those found in the previously reported compound. The azide group is not linear (N3–N2–N1 = 170.84 (11)°) and the least-square line through it forms with the normal to the plane of benzene ring an angle of 46.62 (16)°. The crystal structure is stabilized by C—H···O intermolecular hydrogen bonds (Table 1) forming zig-zag chains parallel to the *b* axis (Fig. 2).

### Experimental

1-*p*-Tolylethanone (8.32 mmol, 1.0 equiv.) was dissolved in acetonitrile (20 ml) in a round bottom flask. To the stirred mixture, *p*-toluene sulphonic acid (12.5 mmol, 1.5 equiv.) and *N*-bromosuccinimide (11.6 mmol, 1.4 equiv.) were added, and the mixtyre refluxed for 1 to 1.5 h until TLC analysis showed no starting material present. The reaction mixture was cooled to room temperature, sodium azide (24.9 mmol, 3.0 equiv.) was added and the mixture further stirred for 2 to 3 hrs followed by the addition of ice cooled water to quench the reaction. The reaction mixture was extracted with ethylacetate (25 ml × 2) and the combined organic layer were dried over anhydrous Na_2_SO_4_, filtered and concentrated in vacuum to get the crude product. The crude product was purified by flash silica gel chromatography (EtOAc/hexane, 1/9–3/7 *v*/*v*) to afford the crystalline title compound in 70% yield. The crystals were found to be suitable for single-crystal X-ray studies. All chemicals were purchased from Sigma-Aldrich.

### Refinement

H atoms were positioned geometrically with C—H = 0.93–0.97 Å, and constrained to ride on their parent atoms with *U*_iso_(H) = 1.2*U*_eq_(C) or 1.5*U*_eq_(C) for methyl H atoms. A rotating group model was applied to the methyl group.

### Crystal data

**Table:**

C_9_H_9_N_3_O	*F*(000) = 368
*M**_r_* = 175.19	*D*_x_ = 1.341 Mg m^−^^3^
Monoclinic, *P*2_1_/*c*	Mo *K*α radiation, λ = 0.71073 Å
Hall symbol: -P 2ybc	Cell parameters from 3299 reflections
*a* = 7.696 (3) Å	θ = 2.8–25.5°
*b* = 9.025 (3) Å	µ = 0.09 mm^−^^1^
*c* = 14.248 (4) Å	*T* = 273 K
β = 118.726 (15)°	Block, colourless
*V* = 867.8 (5) Å^3^	0.30 × 0.21 × 0.17 mm
*Z* = 4	

### Data collection

**Table:**

Bruker SMART APEX CCD area-detector diffractometer	1595 independent reflections
Radiation source: fine-focus sealed tube	1464 reflections with *I* > 2σ(*I*)
Graphite monochromator	*R*_int_ = 0.019
ω scan	θ_max_ = 25.5°, θ_min_ = 2.8°
Absorption correction: multi-scan (*SADABS*; Bruker, 2000)	*h* = −9→9
*T*_min_ = 0.973, *T*_max_ = 0.985	*k* = −10→10
4915 measured reflections	*l* = −16→15

### Refinement

**Table:**

Refinement on *F*^2^	Secondary atom site location: difference Fourier map
Least-squares matrix: full	Hydrogen site location: inferred from neighbouring sites
*R*[*F*^2^ > 2σ(*F*^2^)] = 0.034	H-atom parameters constrained
*wR*(*F*^2^) = 0.090	*w* = 1/[σ^2^(*F*_o_^2^) + (0.0506*P*)^2^ + 0.1892*P*] where *P* = (*F*_o_^2^ + 2*F*_c_^2^)/3
*S* = 1.07	(Δ/σ)_max_ < 0.001
1595 reflections	Δρ_max_ = 0.24 e Å^−^^3^
120 parameters	Δρ_min_ = −0.28 e Å^−^^3^
0 restraints	Extinction correction: *SHELXTL* (Sheldrick, 2008), Fc^*^=kFc[1+0.001xFc^2^λ^3^/sin(2θ)]^-1/4^
Primary atom site location: structure-invariant direct methods	Extinction coefficient: 0.032 (4)

### Special details

**Table:**

Geometry. All e.s.d.'s (except the e.s.d. in the dihedral angle between two l.s. planes) are estimated using the full covariance matrix. The cell e.s.d.'s are taken into account individually in the estimation of e.s.d.'s in distances, angles and torsion angles; correlations between e.s.d.'s in cell parameters are only used when they are defined by crystal symmetry. An approximate (isotropic) treatment of cell e.s.d.'s is used for estimating e.s.d.'s involving l.s. planes.
Refinement. Refinement of *F*^2^ against ALL reflections. The weighted *R*-factor *wR* and goodness of fit *S* are based on *F*^2^, conventional *R*-factors *R* are based on *F*, with *F* set to zero for negative *F*^2^. The threshold expression of *F*^2^ > σ(*F*^2^) is used only for calculating *R*-factors(gt) *etc*. and is not relevant to the choice of reflections for refinement. *R*-factors based on *F*^2^ are statistically about twice as large as those based on *F*, and *R*- factors based on ALL data will be even larger.

### Fractional atomic coordinates and isotropic or equivalent isotropic displacement parameters (Å^2^)

**Table:**

	*x*	*y*	*z*	*U*_iso_*/*U*_eq_	
O1	0.37626 (11)	0.32286 (8)	0.67821 (6)	0.0259 (2)	
N1	0.30617 (13)	0.53889 (11)	0.79105 (8)	0.0250 (3)	
N2	0.46826 (13)	0.49335 (10)	0.86119 (7)	0.0220 (2)	
N3	0.60533 (15)	0.45093 (11)	0.93395 (8)	0.0285 (3)	
C1	0.27074 (15)	0.33313 (12)	0.45933 (9)	0.0225 (3)	
H1B	0.3054	0.2413	0.4929	0.027*	
C2	0.22351 (15)	0.34548 (12)	0.35311 (9)	0.0234 (3)	
H2A	0.2257	0.2615	0.3159	0.028*	
C3	0.17253 (15)	0.48218 (12)	0.30073 (9)	0.0217 (3)	
C4	0.17304 (15)	0.60668 (12)	0.35936 (9)	0.0238 (3)	
H4B	0.1425	0.6991	0.3264	0.029*	
C5	0.21797 (15)	0.59490 (12)	0.46497 (9)	0.0226 (3)	
H5A	0.2157	0.6789	0.5022	0.027*	
C6	0.26701 (14)	0.45736 (11)	0.51680 (9)	0.0198 (3)	
C7	0.31704 (14)	0.43963 (11)	0.63045 (9)	0.0201 (3)	
C8	0.29227 (16)	0.57461 (12)	0.68730 (9)	0.0224 (3)	
H8A	0.3935	0.6468	0.6981	0.027*	
H8B	0.1643	0.6195	0.6418	0.027*	
C9	0.11424 (17)	0.49495 (13)	0.18444 (9)	0.0275 (3)	
H9A	0.1646	0.5861	0.1723	0.041*	
H9B	−0.0276	0.4939	0.1426	0.041*	
H9C	0.1684	0.4130	0.1640	0.041*	

### Atomic displacement parameters (Å^2^)

**Table:**

	*U*^11^	*U*^22^	*U*^33^	*U*^12^	*U*^13^	*U*^23^
O1	0.0310 (4)	0.0219 (4)	0.0239 (4)	0.0038 (3)	0.0125 (4)	0.0038 (3)
N1	0.0204 (5)	0.0313 (5)	0.0230 (5)	0.0014 (4)	0.0103 (4)	−0.0017 (4)
N2	0.0254 (5)	0.0220 (5)	0.0229 (5)	−0.0032 (4)	0.0151 (5)	−0.0038 (4)
N3	0.0296 (5)	0.0333 (6)	0.0225 (5)	0.0008 (4)	0.0125 (5)	0.0017 (4)
C1	0.0214 (5)	0.0199 (5)	0.0245 (6)	0.0039 (4)	0.0097 (4)	0.0022 (4)
C2	0.0226 (5)	0.0233 (6)	0.0239 (6)	0.0029 (4)	0.0109 (4)	−0.0020 (4)
C3	0.0155 (5)	0.0282 (6)	0.0222 (6)	−0.0008 (4)	0.0096 (4)	0.0014 (4)
C4	0.0234 (5)	0.0198 (5)	0.0261 (6)	−0.0005 (4)	0.0104 (5)	0.0045 (4)
C5	0.0227 (5)	0.0187 (5)	0.0246 (6)	−0.0018 (4)	0.0100 (5)	−0.0016 (4)
C6	0.0152 (5)	0.0204 (5)	0.0222 (6)	−0.0010 (4)	0.0076 (4)	−0.0001 (4)
C7	0.0152 (5)	0.0212 (5)	0.0219 (6)	−0.0014 (4)	0.0074 (4)	0.0001 (4)
C8	0.0215 (5)	0.0224 (5)	0.0209 (6)	−0.0002 (4)	0.0083 (4)	−0.0014 (4)
C9	0.0270 (6)	0.0333 (6)	0.0254 (6)	0.0025 (5)	0.0151 (5)	0.0041 (5)

### Geometric parameters (Å, º)

**Table:**

O1—C7	1.2180 (13)	C4—C5	1.3765 (17)
N1—N2	1.2350 (14)	C4—H4B	0.9300
N1—C8	1.4659 (15)	C5—C6	1.4003 (16)
N2—N3	1.1327 (14)	C5—H5A	0.9300
C1—C2	1.3806 (16)	C6—C7	1.4821 (16)
C1—C6	1.3968 (16)	C7—C8	1.5247 (15)
C1—H1B	0.9300	C8—H8A	0.9700
C2—C3	1.3970 (16)	C8—H8B	0.9700
C2—H2A	0.9300	C9—H9A	0.9600
C3—C4	1.3991 (16)	C9—H9B	0.9600
C3—C9	1.4984 (17)	C9—H9C	0.9600
			
N2—N1—C8	116.42 (9)	C1—C6—C7	119.08 (10)
N3—N2—N1	170.84 (11)	C5—C6—C7	122.31 (10)
C2—C1—C6	120.60 (10)	O1—C7—C6	122.33 (10)
C2—C1—H1B	119.7	O1—C7—C8	120.22 (10)
C6—C1—H1B	119.7	C6—C7—C8	117.45 (9)
C1—C2—C3	121.02 (10)	N1—C8—C7	113.13 (9)
C1—C2—H2A	119.5	N1—C8—H8A	109.0
C3—C2—H2A	119.5	C7—C8—H8A	109.0
C2—C3—C4	118.09 (10)	N1—C8—H8B	109.0
C2—C3—C9	121.11 (10)	C7—C8—H8B	109.0
C4—C3—C9	120.79 (10)	H8A—C8—H8B	107.8
C5—C4—C3	121.19 (10)	C3—C9—H9A	109.5
C5—C4—H4B	119.4	C3—C9—H9B	109.5
C3—C4—H4B	119.4	H9A—C9—H9B	109.5
C4—C5—C6	120.49 (10)	C3—C9—H9C	109.5
C4—C5—H5A	119.8	H9A—C9—H9C	109.5
C6—C5—H5A	119.8	H9B—C9—H9C	109.5
C1—C6—C5	118.60 (11)		
			
C6—C1—C2—C3	0.53 (16)	C4—C5—C6—C7	179.79 (9)
C1—C2—C3—C4	0.77 (16)	C1—C6—C7—O1	5.82 (15)
C1—C2—C3—C9	−177.81 (9)	C5—C6—C7—O1	−173.48 (10)
C2—C3—C4—C5	−1.46 (16)	C1—C6—C7—C8	−174.32 (9)
C9—C3—C4—C5	177.13 (10)	C5—C6—C7—C8	6.38 (14)
C3—C4—C5—C6	0.84 (16)	N2—N1—C8—C7	65.13 (12)
C2—C1—C6—C5	−1.16 (15)	O1—C7—C8—N1	−11.81 (14)
C2—C1—C6—C7	179.51 (9)	C6—C7—C8—N1	168.33 (8)
C4—C5—C6—C1	0.48 (16)		

### Hydrogen-bond geometry (Å, º)

**Table:**

*D*—H···*A*	*D*—H	H···*A*	*D*···*A*	*D*—H···*A*
C8—H8*A*···O1^i^	0.97	2.40	3.2404 (19)	145

Symmetry code: (i) −*x*+1, *y*+1/2, −*z*+3/2.
